# The isoleucic acid triad: distinct impacts on plant defense, root growth, and formation of reactive oxygen species

**DOI:** 10.1093/jxb/eraa160

**Published:** 2020-03-28

**Authors:** Sibylle Bauer, Dereje W Mekonnen, Birgit Geist, Birgit Lange, Andrea Ghirardo, Wei Zhang, Anton R Schäffner

**Affiliations:** 1 Institute of Biochemical Plant Pathology, Department of Environmental Sciences, Helmholtz Zentrum München, München, Germany; 2 Institute of Biochemical Plant Pathology, Environmental Simulation Unit, Department of Environmental Sciences, Helmholtz Zentrum München, München, Germany; 3 University of Birmingham, UK

**Keywords:** Arabidopsis, *Brassica napus*, glucosyltransferase, isoleucic acid, plant defense, reactive oxygen species, root growth, salicylic acid, superoxide anion

## Abstract

Isoleucic acid (ILA), a branched-chain amino acid-related 2-hydroxycarboxylic acid, occurs ubiquitously in plants. It enhances pathogen resistance and inhibits root growth of Arabidopsis. The salicylic acid (SA) glucosyltransferase UGT76B1 is able to conjugate ILA. Here, we investigate the role of ILA *in planta* in Arabidopsis and reveal a triad of distinct responses to this small molecule. ILA synergistically co-operates with SA to activate SA-responsive gene expression and resistance in a UGT76B1-dependent manner in agreement with the observed competitive ILA-dependent repression of SA glucosylation by UGT76B1. However, ILA also shows an SA-independent stress response. Nitroblue tetrazolium staining and pharmacological experiments indicate that ILA induces superoxide formation of the wild type and of an SA-deficient (NahG *sid2*) line. In contrast, the inhibitory effect of ILA on root growth is independent of both SA and superoxide induction. These effects of ILA are specific and distinct from its isomeric compound leucic acid and from the amino acid isoleucine. Leucic acid and isoleucine do not induce expression of defense marker genes or superoxide production, whereas both compounds inhibit root growth. All three responses to ILA are also observed in *Brassica napus*.

## Teaser

The 2-hydroxycarboxylic acid isoleucic acid, distinct from closely related compounds, exerts separable SA-dependent and SA-independent responses enhancing plant defense, inducing superoxide formation, and inhibiting root growth.

## Introduction

Several small molecules regulating various processes in plants have been discovered in recent years due to advanced genetic and in particular analytical means. These effectors interact with and diversify canonical plant hormone signaling. Prominent examples are glycerol-3-phosphate, azaleic acid, β-aminobutyric acid, γ-aminobutyric acid, and pipecolic acid. They affect local and systemic defense responses against pathogens, reactions to other stress responses, as well as plant growth and development (Návarová *et al.*, 2012; Yu *et al.*, 2013*a*; Mekonnen *et al.*, 2016; Scholz *et al.*, 2017; Thevenet *et al.*, 2017; Chen *et al.*, 2018; Hartmann *et al.*, 2018; Hartmann and Zeier, 2018). Interestingly, most of them involve primary metabolites such as carbohydrates, lipids, and in particular amino acids. This may indicate an economic utilization of the biosynthetic capacity, but it also constitutes a means to link metabolism and signaling. The isoleucine-related small molecule isoleucic acid (ILA; 2-hydroxy-3-methylpentanoic acid) is another addition to these bioactive small molecules. ILA was discovered in plants by non-targeted metabolome analysis as a substrate of the *Arabidopsis* small-molecule glucosyltransferase UGT76B1. Its exogenous application led to two different phenotypic observations: it enhanced plant resistance against the bacterial pathogen *Pseudomonas syringae* pv. *tomato* and it repressed root growth (von Saint Paul *et al.*, 2011; Maksym *et al.*, 2018). It has remained elusive whether these effects of ILA are linked and how ILA interacts with other signaling pathways. However, it has been suggested that ILA may affect salicylic acid (SA)-dependent pathogen defense by interfering with the SA glucosyltransferase UGT76B1 (Noutoshi *et al.*, 2012; Maksym *et al.*, 2018).

Before its discovery in plants, ILA had been identified in association with maple syrup urine disease of humans, which results in severe brain damage. The disease is due to genetic defects of the degradation pathway of branched-chain amino acids (BCAA) leading to the accumulation of 2-ketocarboxylic acids and the corresponding 2-hydroxycarboxylic acids such as ILA (Mamer and Reimer, 1992; Podebrad *et al.*, 1997; Chuang and Shih, 2001). In plants, ILA is ubiquitously present including monocotyledonous and dicotyledonous plants as well as herbaceous and woody plants, suggesting a universal role of ILA within the plant kingdom. In contrast, the chemically closely related isomeric leucic acid (LA) and the related valic acid have only sporadically been found (Maksym *et al.*, 2018). Hence, the level of ILA and related 2-hydroxyacids seems to be actively controlled and does not appear to be a side-reaction of a constrained BCAA degradation like that in maple syrup urine disease (Maksym *et al*., 2018).

SA is a key signaling molecule in plants involved in developmental processes, response to abiotic stresses, and, importantly, inducing and organizing pathogen defense. The endogenous SA is induced upon perception of bacterial pathogens. The initial formation of apoplastic superoxide anions and hydrogen peroxide triggers a cascade leading to enhanced biosynthesis of SA. It is assumed that SA and H_2_O_2_ are engaged in a self-amplifying feedback loop (Torres *et al.*, 2006; Vlot *et al.*, 2009; Herrera-Vásquez *et al.*, 2015; Mignolet-Spruyt *et al.*, 2016; Dempsey and Klessig, 2017; Waszczak *et al.*, 2018), and therefore mitigating processes are crucial to control the signal and the corresponding responses. In addition to catabolism, the most important SA attenuating reaction is conjugation of SA to form SA-*O*-glucoside (SAG) or SA glucose ester. In *Arabidopsis*, UGT76B1 has been characterized as a SA glucosyltransferase generating SAG (von Saint Paul *et al.*, 2011; Noutoshi *et al.*, 2012; Li *et al.*, 2015). Loss-of-function *ugt76b1* mutants are more resistant to the bacterial pathogen *P. syringae* pv. *tomato* and they constitutively enhance transcription of SA pathway marker genes, e.g. *PR1*. This phenotype is related to the loss of UGT76B1-dependent SA glucosylation. However, UGT76B1 also conjugated ILA, and ILA inhibited SA glucosylation *in vitro*. This suggests that ILA may interfere with SA homeostasis or signaling (von Saint Paul *et al.*, 2011; Noutoshi *et al.*, 2012).

Apart from its impact on pathogen defense, ILA represses root growth (von Saint Paul *et al.*, 2011). Root growth is sensitive to many cues ranging from water and nutrient availability to stress responses. Exogenous application of several plant hormones such as auxin, cytokinin, jasmonic acid, or SA represses root growth (Xia *et al.*, 2015; Tsukagoshi, 2016). Changes in the formation of hydrogen peroxide, superoxide anions, or hydroxyl radicals and their spatial distribution as well as alterations of the antioxidants glutathione and thioredoxins are frequently involved in these responses, but also in controlling regular root development (Gapper and Dolan, 2006; Dunand *et al.*, 2007; Xia *et al.*, 2015; Tsukagoshi, 2016).

Here, we examined whether and how ILA functionally interacts with SA in pathogen defense responses and in root growth inhibition. Since ROS are involved in plant defense responses and in root growth, we also analysed the involvement of ILA-dependent ROS formation in both contexts. Furthermore, ILA was compared with LA and the amino acid isoleucine to assess the specificity of its actions. Thereby, ILA was established as a small molecule regulating three separable processes in SA-dependent and SA-independent as well as in ROS-dependent and ROS-independent manners. These effects of ILA are conserved at least within the Brassicaceae family, since *Brassica napus* exhibited similar ILA responses as *Arabidopsis*.

## Materials and methods

### Plant materials

Several *Arabidopsis* mutants or genetic crosses thereof were used in addition to the wild type (WT, accession Col). Mutant lines were obtained from the *Arabidopsis* stock centers (Scholl *et al.*, 2000; Sessions *et al.*, 2002; Alonso *et al.*, 2003) unless otherwise indicated. These included the *ugt76b1-1* knockout mutant (AT3G11340; SAIL_1171A11; von Saint Paul *et al.*, 2011), *sid2-1* (AT1G74710; Nawrath and Métraux, 1999), *rbohd* (SALK_070610; AT5G47910; Pogány *et al.*, 2009), *rbohf* (SALK_059888, AT1G64060; Pogány *et al.*, 2009), *abi1-2* (SALK_072009, AT4G26080; Wang *et al.*, 2018), *aba2-1* (N156; AT1G52340; Christmann *et al.*, 2005), *jar1-1* (N8072, AT2G46370; Staswick *et al.*, 2002), *jin1/myc2* (AT1G32640; Berger *et al.*, 1996), and *ein2-1* (N65994, AT5G03280; Guzmán and Ecker, 1990). In addition, a line constitutively overexpressing UGT76B1 (von Saint Paul *et al.*, 2011) and a transgenic line expressing the bacterial *nahG* (Gaffney *et al.*, 1993) were employed. Multiple mutants were established by the corresponding genetic crosses. The *Brassica napus* PBY0180 Darmor line was obtained from the Genebank at IPK Gatersleben (Gatersleben Germany). All experiments were carried out in a controlled growth chamber (light/dark regime 10/14 h at 20/16 °C, 65/80% relative humidity, light at 130 µmol m^−2^ s^−1^) of photosynthetic photon flux density. Plants were grown on a peat moss-based substrate (Floragard Multiplication substrate, Oldenburg, Germany)–quartz sand (8:1) mixture, in liquid medium or on plates containing solidified medium as specified.

### Bacterial infection

For pathogen infections, *Arabidopsis* plants were grown on peat moss substrate in 80 ml pots for 28 d under a short-day, 10 h light regime. In cases of chemical treatment, 10 ml of 10 µM SA or 250 µM ILA solution or the combination thereof (pH adjusted to 5.7) was used to water these plants. After 3 d, leaves were inoculated by syringe-infiltration with *P. syringae* pv. *tomato* DC3000 (5×10^5^ colony-forming units (cfu) ml^−1^ in 10 mM MgCl_2_). Three leaf discs from three plants per replicate were harvested 72 h after infection to determine bacterial titers (cfu cm^−2^). In a separate experiment, the uptake of ILA to the rosette leaves was confirmed. Three days after watering with 250 µM ILA, the internal level of ILA was determined (Maksym *et al.*, 2018). The level was raised from 46±2.5 nM (control) to 5.5±1.0 µM after ILA application (mean ±SE; *n*=4).

### Metabolite analyses

ILA was quantified by gas chromatography coupled to mass spectrometry after extraction from freeze-dried plant material (Maksym *et al.*, 2018).

### Chemical treatments in liquid culture

Seeds were surface sterilized with 80% ethanol and 30% commercial bleach, and finally washed four times with sterile water. After cold treatment (4 °C) for 2–3 d, plants were grown with 10 h light for 14 d in six-well dishes on a shaker at 100 rpm. Wells contained 5 ml half-strength Murashige and Skoog medium (Duchefa Biochemie, Haarlem, The Netherlands; pH 5.7) containing 1% sucrose (Roth, Karlsruhe, Germany). After 12 d, media were replaced with fresh medium or with medium containing the indicated concentration of SA (Sigma-Aldrich, München, Germany), ILA (Interchim, Montluçon, France), isoleucine (Ile; Sigma-Aldrich) and/or LA (Sigma-Aldrich). Four millimolar 4-hydroxy-2,2,6,6-tetramethylpiperidine 1-oxyl (4-OH-TEMPO; Sigma-Aldrich) was added to scavenge superoxide radicals (Yokawa *et al.*, 2011). Ten micromolar diphenyleneiodonium chloride (DPI; Sigma-Aldrich) was used to block the activity of NADPH oxidases (Yokawa *et al.*, 2011). All solutions were adjusted to pH 5.7 and filter-sterilized before use. Plants were harvested after the indicated time for further analyses; for RNA isolation and metabolic measurements only leaf samples washed with water were used. Five hundred micromolar ILA was used to test effects triggered by ILA alone, whereas 250 µM ILA was employed to analyse interactive effects of SA and ILA, since the lower ILA concentration triggered less pronounced or no significant effects on its own.

The uptake of ILA was confirmed by analysing rosette leaves treated with 250 µM ILA; the average internal concentration was 9.3±1.7 µM in comparison with 0.19±0.05 µM in untreated samples (mean ±SE; *n*=4).

### Root growth assay on plates

*Arabidopsis* or *Brassica napus* seeds were surface sterilized and grown on plates with half-strength Murashige and Skoog medium, 0.5% Gelrite (Duchefa Biochemie) and 1% sucrose (Roth, Karlsruhe, Germany). Seeds were transferred to square Petri dishes containing no additives or different concentrations of SA, ILA, Ile, LA, and/or LA. The pH of the growth medium was adjusted to 5.7–5.8 with KOH. Four millimolar 4-OH-TEMPO was included to scavenge superoxide radicals (Yokawa *et al.*, 2011). After cold treatment (4 °C) for at least 2 d, plants were grown under short day conditions for 9–10 d (*Arabidopsis*) and 9 d (*B. napus*). Root length was analysed using ImageJ (version 1.51w). Root cells were imaged using confocal microscopy (Leica SP8, Wetzlar, Germany) after staining of seedlings in 1 mM propidium iodide for 2 min. The lengths of meristems and the cell lengths in the differentiation zone were analysed using ImageJ.

### Reactive oxygen species staining

*Arabidopsis* plantlets were vacuum infiltrated with 0.1% (w/v) nitroblue tetrazolium (NBT; Sigma-Aldrich) in 50 mM potassium phosphate (pH 6.4), 10 mM NaN_3_ and incubated in the dark for 2 min (roots) and 5 min (leaves) to visualize superoxide production. *Brassica napus* plants were grown for 9 d on control or 500 µM ILA plates; roots were harvested, vacuum infiltrated with NBT staining solution, and directly stopped for destaining. Pixels of the images of single leaves or roots were analysed using ImageJ (version 1.51w) with the ImageJ-based macro PIDIQ by applying a blue spectrum filter (hue: 140–190) (Laflamme *et al.*, 2016). These data were used for a semi-quantitative assessment of the NBT staining. H_2_O_2_ formation was addressed by 3,3′-diaminobenzidine (DAB) staining (Daudi and O’Brien, 2012). Roots were vacuum infiltrated with 1 mg ml^−1^ DAB tetrahydrochloride (pH>6.5) (Sigma-Aldrich) and afterwards incubated for 1 h in the dark, whereas leaves were incubated for 8 h in DAB solution. In all cases, chlorophyll was removed with ethanol at 80 °C. Images were taken using an Olympus BX61 microscope (Olympus, Hamburg, Germany).

### Quantitative real-time PCR

Total RNA extraction was carried out with innuPREP RNA Kit (Analytik Jena, Jena, Germany). One microgram of total RNA was reverse-transcribed using QuantiTect Reverse Transcription Kit (Qiagen, Hilden, Germany). Real-time PCR (qRT-PCR) quantification was performed using gene-specific oligonucleotides (see Supplementary Table S1 at *JXB* online) and SensiMix™ SYBR Low-ROX (Bioline, Luckenwalde, Germany) in duplicate assays (7500 real-time PCR system, Applied Biosystems/Thermo Fisher Scientific, Dreieich, Germany). *UBQ5* and *S16* were used to normalize expression values (Vandesompele *et al.*, 2002) as *normalized relative quantities* (NRQ). *Brassica napus UP1* and *UBQ9* were used as reference genes for canola samples (Chen *et al.*, 2010). Cycle values and efficiency of reaction were extracted from the raw data with the qPCR package (Spiess, 2018) and NRQs were calculated by Microsoft Excel (Hellemans *et al.*, 2007).

### Statistical analyses

Statistical analyses were performed using R (R 3.5.1 for Windows). For robust statistical analyses, the WRS2 package based on Wilcox’s WRS functions was used. Two groups were compared with Welch’s two-sample *t*-test. One-way multiple group comparisons were tested in R using the robust one-way ANOVA function t1way with lincon *post hoc* test and *P*-values were Holm-corrected. Two-way ANOVA was performed with the t2way function in R (Mair and Wilcox, 2018). Two-way ANOVA with treatment and time as discrete factors and the Tukey method for all pairwise multiple comparisons were used to analyse dynamics in root growth after log transformation of root length measurements.

## Results

### Exogenous isoleucic acid enhances salicylic acid-dependent plant defense to biotrophic pathogen

**Fig. 1.**
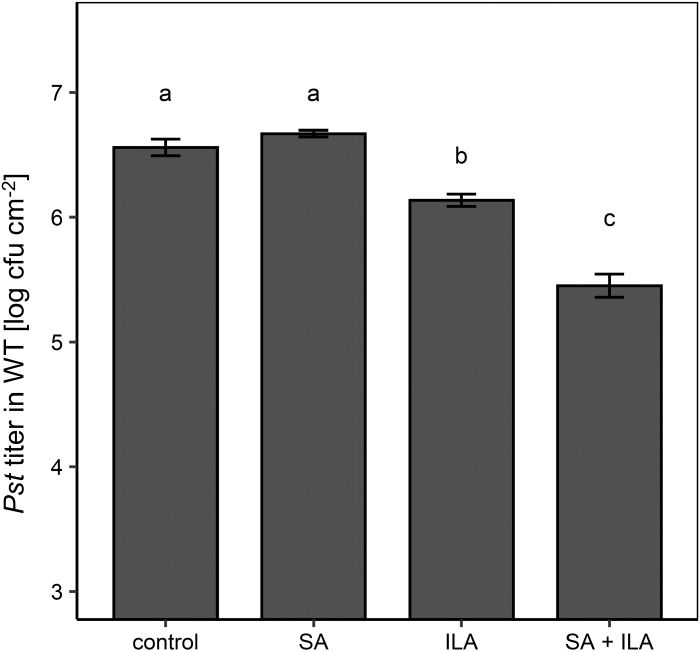
Repression of bacterial pathogens upon SA and ILA application. Four-week-old soil-grown plants were watered with 10 µM SA, 250 µM ILA, the combination of 10 µM SA and 250 µM ILA, or without any addition (control). After 3 d the plants were inoculated with *Pseudomonas syringae* pv. *tomato* DC3000 (5×10^5^ cfu ml^−1^), and the resulting *P. syringae* titers were determined 3 d after infection. Bars represent the means ±SE of four biological replicates. Significant differences (*P*_adj_ values) are indicated by letters according to one-way ANOVA. The experiment was independently repeated three times with similar results.

The competitive inhibition of ILA on UGT76B1-catalysed SA glucosylation *in vitro* as well as the induction of pathogen resistance by exogenous ILA application (von Saint Paul *et al.*, 2011; Noutoshi *et al.*, 2012; Supplementary Fig. S1) raised the question of whether and how ILA interferes with SA signaling *in vivo*. To address this interaction during plant defense, soil-grown wild type plants were watered with 10 µM SA, 250 µM ILA, and a combination thereof before infection by virulent *P. syringae* DC3000. A low-concentration, 10 µM SA solution did not affect a subsequent *Pseudomonas* infection in comparison with mock-watered plants (Fig. 1). Similarly, treatment with 250 µM ILA alone did not significantly reduce bacterial growth. In contrast, the combination of 10 µM SA and 250 µM ILA provoked an enhanced resistance and repressed bacterial growth more than 10-fold relative to the control or SA watering (Fig. 1).

### Isoleucic acid-enhanced defense response requires salicylic acid and UGT76B1

**Fig. 2.**
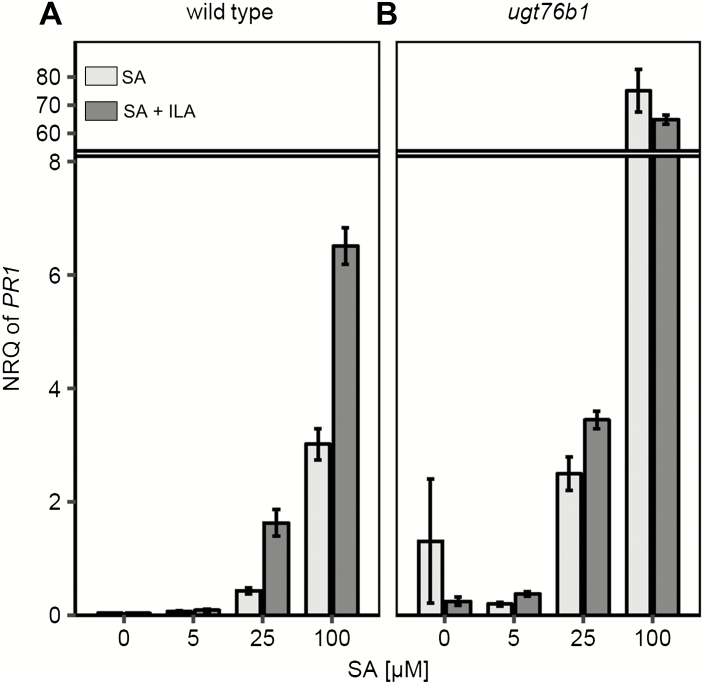
Interaction of SA and ILA *in planta. PR1* expression in wild type (A) and *PR1* expression in *ugt76b1* (B) was investigated in leaves of 12-day-old seedlings that had been incubated 48 h with increasing concentrations of SA (0, 5, 25, 100 µM) in the absence (light-grey bars) and presence (dark grey bars) of 250 µM ILA. Expression values are normalized to *S16* and *UBQ5* (NRQ, normalized relative quantity); means ±SE; *n*=3–4.

Plants were treated with 250 µM ILA in combination with rising, yet low concentrations of SA in order to probe the interaction of SA and ILA in pathogen defense at the molecular level. In line with the pathogen susceptibility, 250 µM ILA alone induced *PR1* transcription only weakly, whereas 500 µM showed a more robust response (Fig. 2; Supplementary Fig. S2A). In the absence of ILA, the expression of *PR1* was gradually induced with increasing concentrations of SA. However, when ILA and SA were combined, a reinforced response of the SA-induced *PR1* expression was observed (Fig. 2A). A two-way between-groups analysis of variance revealed a significant impact of SA and ILA on *PR1* expression and a significant interaction term indicative of a non-additive, positive interaction (*P*<0.005 for both factors and for the interaction term). A similar effect was observed when SA levels were raised to 750 µM in combination with 250 µM ILA. Thus, the ILA enhancement of the SA response was still effective (see Supplementary Fig. S3A). The converse experiment, i.e. the addition of increasing concentrations of ILA to a fixed level of SA, confirmed the positive interaction of ILA and SA and showed an ILA concentration-dependent up-regulation of *PR1* expression up to 500 µM ILA in combination with 100 µM SA (Supplementary Fig. S3B).

Next, we were interested to determine the role of UGT76B1 in the interaction of ILA and SA during defense responses, since both compounds are substrates of UGT76B1 *in vitro*. SA glucosylation attenuates the SA response (Vlot *et al.*, 2009). Therefore, we hypothesized that ILA glucosylation may compete with the UGT76B1-dependent SA inactivation, thereby fostering the SA-dependent defense response (Supplementary Fig. S1; Noutoshi *et al.*, 2012). To test that hypothesis, *ugt76b1* seedlings were treated with SA alone and in combination with ILA. Similar to the wild type, *ugt76b1* also showed an increasing expression of *PR1* upon application of rising exogenous SA, although at a much more pronounced level. However, in contrast to the wild type, a further enhancement of *PR1* expression by the additional application of 250 µM ILA was not observed (Fig. 2B). Two-way between-groups analysis of variance showed only an influence of the SA concentration (*P*<0.01) on *PR1*, whereas the effect of ILA (*P*=0.269) and the interaction (*P*=0.137) were not significant. The expression of *PR1* was induced to an even higher relative level, with higher SA concentrations demonstrating that the *PR1* induction did not plateau when 100 µM SA was applied in combination with 250 µM ILA (Fig. 2B; Supplementary Fig. S4). In addition, the ILA-dependent up-regulation of *PR1* expression required SA, since SA-depleted NahG *sid2* plants did not show this enhancement even after application of 500 µM ILA (see Supplementary Fig. S2B). Thus, the ILA-enhanced defense response was dependent on UGT76B1 and SA.

### Isoleucic acid induces superoxide anion formation

**Fig. 3.**
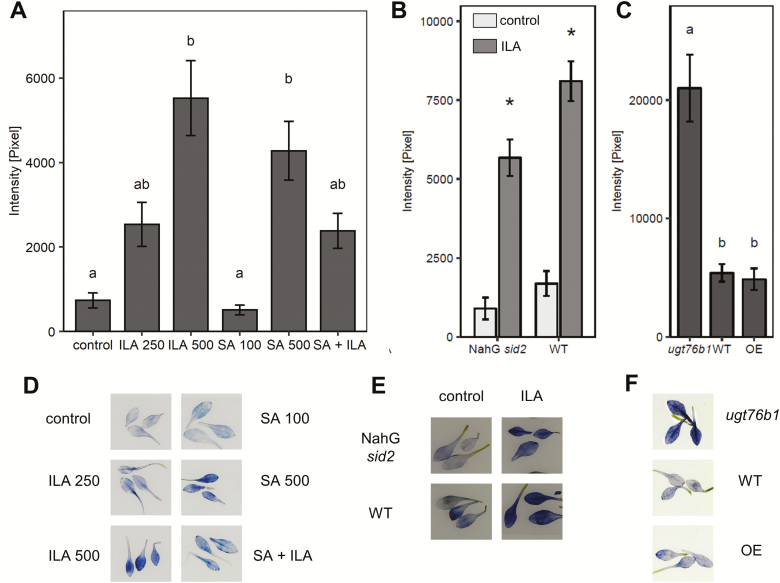
ILA enhances superoxide radicals in leaves. NBT staining in leaves of 2-week-old seedlings was assessed as a semi-quantitative measurement of O_2_^−^ formation (compare Fig. 4A, B). (A) NBT staining 48 h after treatment with 250 µM ILA, 500 µM ILA, 100 µM SA, 500 µM SA, or the combination of 100 µM SA + 250 µM ILA. Means ±SE; *n*=9. Significant differences (*P*_adj_<0.05) are indicated by letters according to one-way ANOVA. (B) NBT staining in 2-week-old NahG *sid2* (*n*=21) and wild type (WT; *n*=15) seedlings 48 h after treatment with 500 µM ILA. Means ±SE; differences between treated or untreated plants were analysed by Welch’s two sample *t*-test. (C) NBT staining detected in leaves of *ugt76b1*, wild type (WT) and UGT76B1 overexpressor (OE). Means ±SE; *n*=20–23. Significant differences (*P*_adj_<0.05) are indicated by letters according to one-way ANOVA. The experiments were independently repeated three times with similar results. (D–F) Representative images of NBT staining of leaves of the indicated genotypes and treatments, which were analysed in (A), (B), and (C), respectively.

**Fig. 4.**
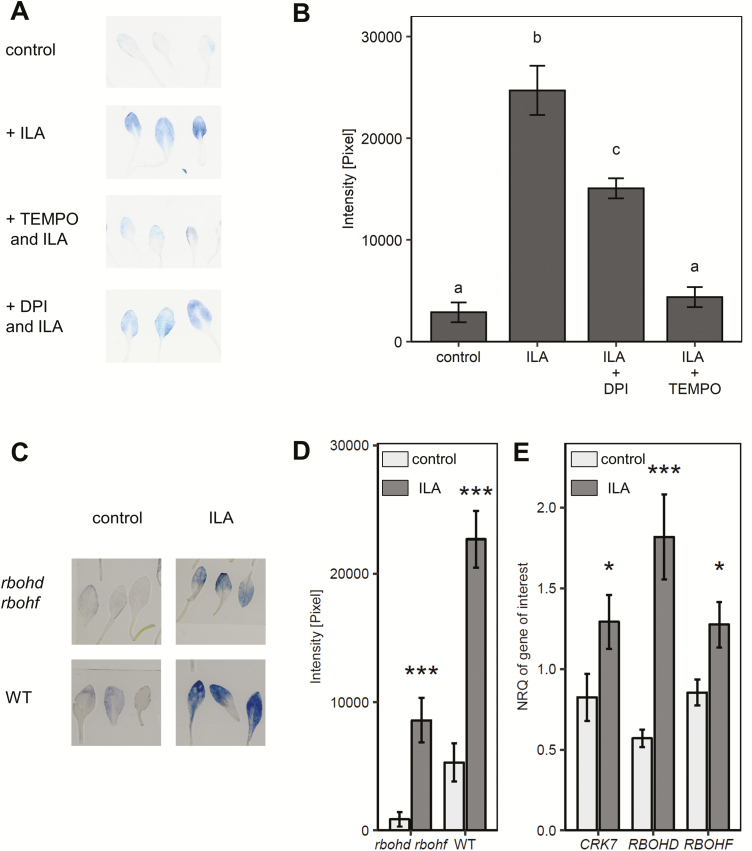
NADPH oxidases contribute only partially to ILA-induced superoxide formation in leaves. (A, B) NBT staining is sensitive to superoxide scavenger 4-OH-TEMPO (TEMPO) and reduced by addition of DPI. Twelve-day-old seedlings were treated with 500 µM ILA, with ILA and 4-OH-TEMPO, or with ILA and DPI for 3.5 h. NBT staining of the leaves was determined as a semi-quantitative measurement. Means ±SE; *n*=9. Significant differences (*P*_adj_<0.05) are indicated by letters according to one-way ANOVA. (C) Fourteen-day-old wild type seedlings treated for 48 h either with control medium (light grey bars) or with medium containing 500 µM ILA (dark grey bars). ROS-related genes (*CRK7*, *RBOHD*, and *RBOHF*) were induced by exogenous ILA application in wild type. Gene expression was assessed by RT-qPCR and normalized to *S16* and *UBQ5*. Means ±SE; *n*=4; differences between treated or untreated plants were analysed by Welch’s two sample *t*-test. *P<0.05, ***P<0.001. (D, E) O_2_^−^ radical detected by NBT staining in 14-day-old wild type and *rbohd rbohf* seedlings treated for 48 h either with control medium (light grey bars) or with medium containing 500 µM ILA (dark grey bars). Means ±SE; *n*=9. Differences between treated and untreated plants were analysed by Welch’s two sample *t*-test. *P<0.05, ***P<0.001.

Reactive oxygen species (ROS) are intrinsically involved in SA-dependent defense reactions and they can be induced by exogenous application of SA (Torres *et al.*, 2006; Vlot *et al.*, 2009; Khokon *et al.*, 2011; Herrera-Vásquez *et al.*, 2015). The impact of ILA on ROS production was examined, since ILA enhances SA-related responses (Figs 1, 2). *Arabidopsis* seedlings were treated with ILA, SA, and a combination of both compounds and stained with NBT to detect O_2_^−^ radical formation. Five hundred micromolar ILA induced similar NBT staining as 500 µM SA 48 h after the application of the chemicals (Fig. 3A, D). Since NBT is not specific for detecting superoxide anions, we employed the superoxide scavenger 4-OH-TEMPO (Yokawa *et al.*, 2011; Noctor *et al.*, 2016). Indeed, 4-OH-TEMPO suppressed the NBT signal supporting the formation of superoxide (Fig. 4A, B). Since ROS induction is one of the earliest cellular responses following pathogen recognition (Torres *et al.*, 2006), earlier time points after ILA application (3 and 24 h *vs.* 48 h) were also examined. Enhancement of O_2_^−^ radicals due to the application of ILA was both a rapid and a sustainable response (see Supplementary Fig. S5A). In contrast, DAB staining of wild type plants grown in the presence of 500 µM ILA did not reveal enhanced H_2_O_2_ content in leaves (Supplementary Fig. S5B).

Again 100 µM SA and 250 µM ILA alone and in combination were used to test a potential interaction of SA and ILA in superoxide induction. In contrast to *PR1* gene expression (Fig. 2A), 100 µM SA did not evoke a detectable effect on O_2_^−^ formation, whereas 250 µM ILA resulted in an enhanced superoxide production; NBT staining was even further increased by 500 µM ILA (Fig. 3A). Interestingly, the combined application of 100 µM SA and 250 µM ILA did not show a further enhancement and thereby differed from the observed interaction in the defense response (Fig. 3A, D). Thus, ILA may induce superoxide formation independent of SA. To test that hypothesis, the response of NahG *sid2* and wild type plants to ILA was compared; 500 µM ILA were employed to induce a stronger response (Fig. 3A). Intriguingly, ILA treatment induced NBT staining in both lines suggesting an SA-independent O_2_^−^ induction (Fig. 3B, E). To further investigate the link between endogenous ILA levels and superoxide production, a UGT76B1-overexpression line, wild type, and the *ugt76b1* loss-of-function mutant were analysed. These lines contain increasing levels of unconjugated ILA due to the presence or absence of the glucosyltransferase (Maksym *et al.*, 2018). Intriguingly, the *ugt76b1* knockout mutant containing the highest endogenous ILA level exhibited enhanced constitutive O_2_^−^ production as compared with the wild type and the UGT76B1 overexpressor (Fig. 3C, F). The enhanced superoxide production has also been found in the SA-depleted NahG *sid2 ugt76b1* triple mutant, confirming its independence of SA (see Supplementary Fig. S5C).

The NADPH oxidases RESPIRATORY BURST OXIDASE HOMOLOGUES D and F (RBOHD and RBOHF) are key components and crucial for apoplastic ROS production in response to pathogen attack (Morales *et al.*, 2016). To address their involvement in ILA-induced superoxide formation, we first examined the changes in *RBOHD* and *RBOHF* transcripts upon treatment with 500 µM ILA. The expression of *RBOHD* was induced 3-fold, while *RBOHF* was only slightly up-regulated (Fig. 4C). We also examined the expression of *CRK7*, a known mediator of oxidative signaling induced by extracellular ROS (Idänheimo *et al.*, 2014) and found that its expression was significantly induced (Fig. 4A). Next, we examined the ability of the double knockout mutant *rbohd rbohf* to produce O_2_^−^ radicals after application of 500 µM ILA. ILA was still able to induce O_2_^−^ radicals in the *rbohd rbohf* double mutant, but to a lesser extent than in wild type (Fig. 4D, E). There was still a significant induction of NBT staining when DPI, an inhibitor of flavin-containing enzymes and general NAPDH oxidase inhibitor, was applied together with ILA, indicating the independence of ILA-induced ROS from NADPH oxidases and the involvement of other superoxide-producing components (Fig. 4A, B).

### Root growth is inhibited by salicylic acid and isoleucic acid

**Fig. 5.**
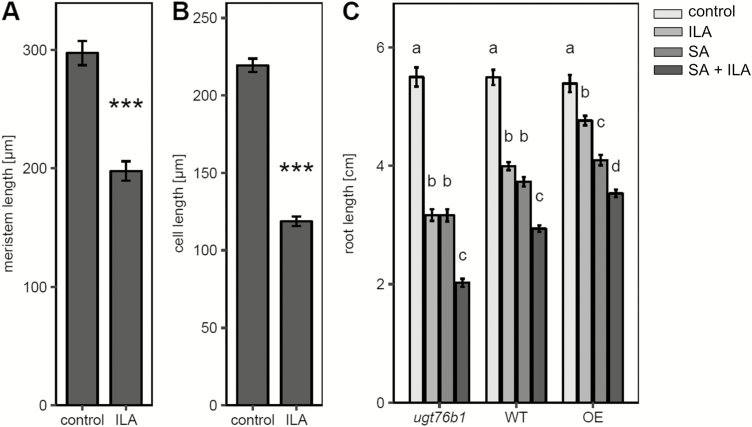
Root growth inhibition by ILA. (A) Root meristem length of 8-day-old seedlings grown on plates with 500 µM ILA or control medium. Means ±SE; *n*=17 (control), 15 (ILA). (B) Longitudinal extension of epidermal cells in the root differentiation zone of the seedlings grown as in (A). Means ±SE; *n*=257 (control), 197 (ILA). (C) Root length of *ugt76b1*, wild type, and UGT76B1 overexpressor (OE) plants grown on control medium and on medium containing 250 µM ILA, 10 µM SA, or the combination of ILA and SA for 10 d. Means ±SE; *n*=21–30. Significant differences (*P*_adj_<0.05) are indicated by letters according to one-way ANOVA assessed for the genotypes. The experiments were independently repeated three times with similar results.

Exogenous ILA represses root growth in addition to the effect on pathogen defense (von Saint Paul *et al.*, 2011). To substantiate the ILA-induced shortening of roots at the cellular level we examined root meristem size and root cell elongation. ILA treatment reduced the meristem size and the sizes of root epidermal cells indicating repressive effects on both meristem development and cell elongation (Fig. 5A, B). Interestingly, ILA repressed root growth independently of UGT76B1, since the inhibition had also been observed for the *ugt76b1* loss-of-function mutant (Fig. 5C). Thus, UGT76B1 was not required in establishing this root phenotype in contrast to the defense-related ILA response. The inhibition was even more pronounced in *ugt76b1* as compared with the wild type, whereas it was attenuated in the UGT76B1-overexpressing line (Fig. 5C).

**Fig. 6.**
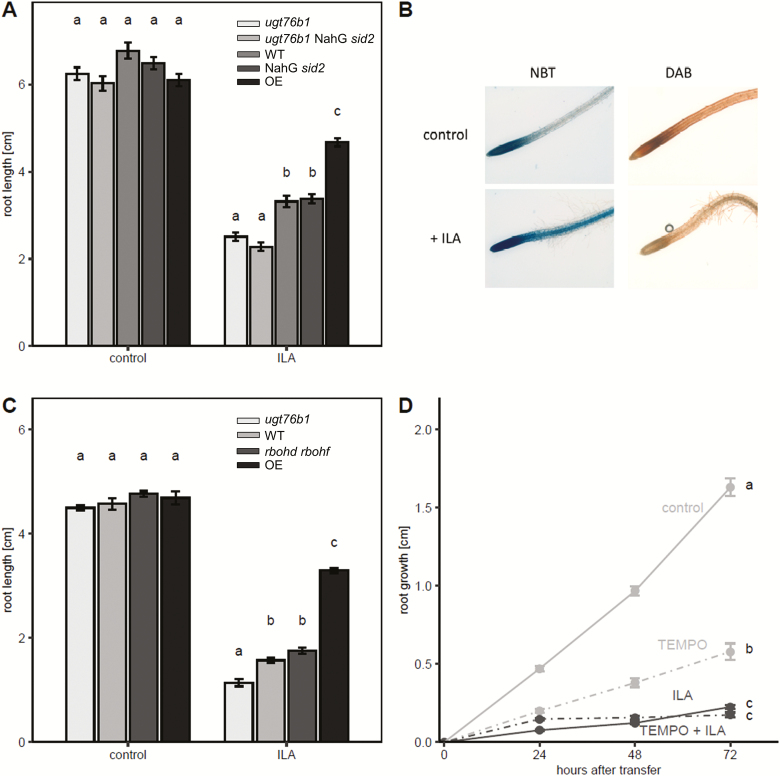
SA- and superoxide-independent root growth inhibition by ILA. (A) Root growth inhibition on media without (control) or with 500 µM ILA for *ugt76b1*, *ugt76b1* NahG *sid2*, wild type, NahG *sid2*, and UGT76B1 overexpressor (from left to right, light grey to black bars) after 10 d. (B) NBT and DAB staining of primary root tips of wild type plants after growth on control or 500 µM ILA plates after 10 d. (C) Root length of 9-day-old *ugt76b1*, wild type, *rbohd rbohf*, and UGT76B1 overexpressor plants (from left to right, light grey to dark grey bars) on media without (control) or with 500 µM ILA. Significant differences among the genotypes of each group in (A) and (C) (*P*_adj_<0.05) are indicated by letters according to one-way ANOVA. Means ±SE; *n*=13–18. (D) ILA-induced root growth inhibition in the presence of the O_2_^−^ scavenger 4-OH-TEMPO. Means ±SE; *n*=10–32. Different lowercase letters indicate a significant difference according to a two-way ANOVA with treatment and time as discrete factors (*P*_adj_<0.05). The experiment was independently repeated two times.

SA also inhibits root growth at relatively low concentrations (Wildermuth *et al.*, 2001). Therefore, we examined whether there is an interaction of SA and ILA in establishing the root phenotype. In the wild type, 10 µM SA and 250 µM ILA provoked similar root growth inhibition, whereas the combination of both compounds led to a further significant reduction of root growth. The pattern was also evident in *ugt76b1* and the UGT76B1-overexpressing line (Fig. 5C). The SA-deficient NahG *sid2* was employed to explore whether the ILA-related root growth inhibition was dependent on SA. However, the root growth response of NahG *sid2* and NahG *sid2 ugt76b1* was similar to the reaction of the wild type and *ugt76b1*, respectively (Fig. 6A), indicating that the inhibition of root growth by ILA was independent of SA. Similarly, mutants affecting abscisic acid (ABA) biosynthesis and perception, jasmonic acid (JA) perception and signaling, or ethylene signaling did not abolish the root growth inhibition by ILA (see Supplementary Fig. S6).

Since ROS and their relative spatial distribution are involved in regulation of root growth (Dunand *et al.*, 2007; Tsukagoshi, 2016), the formation of O_2_^−^ and H_2_O_2_ in roots in response to exogenous ILA was assessed. H_2_O_2_ levels detected by DAB staining did not increase, whereas NBT staining indicated an ILA-enhanced superoxide formation in the elongation zone and in the meristem (Fig. 6B). Enhanced and spatially extended superoxide production by the interplay of NADPH oxidases and peroxidases was correlated to larger meristems and increased cell elongation (Tsukagoshi, 2016), whereas the addition of ILA led to enhanced superoxide production and reduced meristem size. Therefore, the relation of superoxide formation and root growth inhibition was explored. A wild type-like repression of root growth was observed, when the *rbohd rbohf* double mutant was challenged with 500 µM ILA (Fig. 6C). Furthermore, we employed 4-OH-TEMPO to analyse whether the root growth inhibition by ILA was affected by scavenging superoxide anions (Yokawa *et al.*, 2011). Root growth was suppressed by ILA in the presence of the superoxide scavenger (Fig. 6D). Thus, both the genetic analysis and the pharmacological approach supported the independence of the ILA-provoked root growth inhibition from ILA-induced O_2_^−^.

### The effects of the structurally related leucic acid and isoleucine are distinct from isoleucic acid-induced effects

**Fig. 7.**
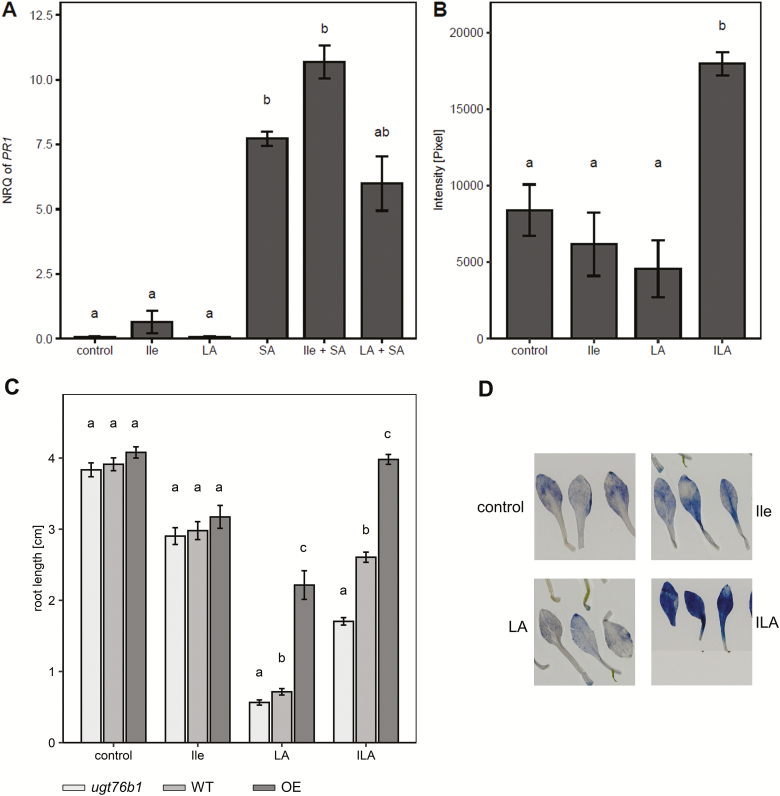
LA and Ile do not affect SA signaling and ROS induction, but also show a root inhibition effect. (A) *PR1* expression in response to 500 µM Ile, 500 µM LA, and 100 µM SA as well as to the combined treatments with 250 µM Ile + 100 µM SA and 250 µM LA + 100 µM SA. *PR1* expression was determined by RT-qPCR and normalized to *S16* and *UBQ5*. Means ±SE; *n*=3–4. (B) Superoxide radical induction assessed by NBT staining 48 h after application of 500 µM Ile, LA, or ILA to 2-week-old seedlings (*n*=12). The experiment was independently repeated two times with similar results. (C) Root growth inhibition on ILA-, Ile-, and LA-containing media; a lower level of 250 µM each was used for this comparison of individually applied compounds, since 250 µM LA exerted already a very strong effect (Supplementary Fig. S7). Root length was recorded after 9 d. Data for *ugt76b1* (light grey), wild type (grey), and UGT76B1 overexpressor (dark grey) were compared within the treatments; *n*=19–23. The experiment was independently repeated two times with similar results. Significant differences (*P*_adj_<0.05) are indicated by letters according to one-way ANOVA. (D) Representative NBT-stained leaves after treatment with Ile, LA, and ILA.

LA (related to ILA by shifting the methyl side group of one position) and isoleucine (exchanging the 2-hydroxyl group of ILA for an amino group) were examined to elucidate whether endogenously occurring compounds structurally related to ILA provoke similar effects. Seedlings were treated with LA or Ile alone and in combination with SA. *PR1* expression was not significantly affected by the addition of LA or Ile (Fig. 7A). Furthermore, O_2_^−^ staining was not enhanced by the application of either LA or Ile (Fig. 7B, D). In contrast, all compounds reduced root growth with Ile being the least effective and LA having the strongest impact (Fig. 7C; Supplementary Fig. S7). Interestingly, Ile-related growth repression was independent of the UGT76B1 expression level, whereas both ILA and LA showed a UGT76B1*-*dependent pattern. Wild-type or constitutive UGT76B1 expression mitigated the root growth inhibition (Fig. 7C), which may be attributed to the ability of UGT76B1 to glucosylate ILA and LA as shown *in vitro* using recombinant UGT76B1 (Maksym *et al.*, 2018).

### Isoleucic acid responses are conserved in *Brassica napus*

**Fig. 8.**
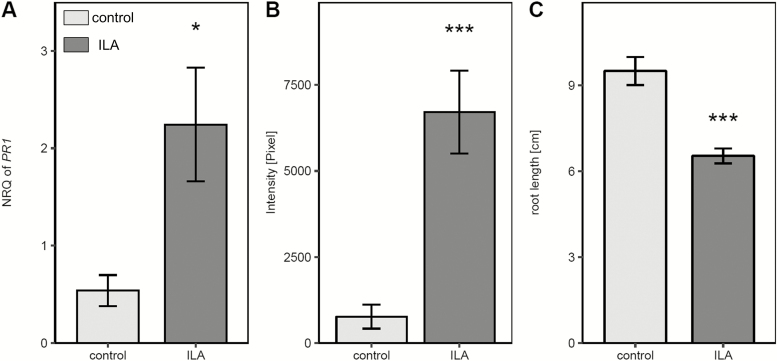
*PR1* expression, ROS induction, and root growth inhibition upon ILA treatment in *Brassica napus.* Seedlings were grown for 9 d on either control medium (light grey bars) or medium containing 500 µM ILA (dark grey bars). (A) *BnPR1* expression level in *B. napus* leaves was assessed by RT-qPCR and normalized to *BnUP1* and *BnUBQ9*. Means ±SE, *n*=3. (B) NBT staining of *B. napus* roots. Means ±SE, *n*=15. (C) Root length of *B. napus* plants. Means ±SE, *n*=10–11. Welch’s two sample *t*-test was performed to test differences between untreated and treated plants; *P<0.05, ***P<0.001.

ILA as well as LA has been detected in other plant species including members of the Brassicaceae. To assess whether ILA might act in a similar manner in crop species, *Brassica napus* seedlings were tested for ILA-induced *PR* gene expression, superoxide formation, and root growth inhibition. *BnPR1* was induced in leaves when seedlings were grown on 500 µM ILA-containing plates (Fig. 8A). In roots of these plants O_2_^−^ radicals were strongly enhanced in comparison with control plants (Fig. 8B). Eventually, ILA was effective in reducing root growth in *B. napus* (Fig. 8C). Thus, for all phenotypes, the response of *B. napus* to the small-molecule effector ILA was similar to *Arabidopsis*.

## Discussion

### Isoleucic acid activation of salicylic acid-dependent defense responses

**Fig. 9.**
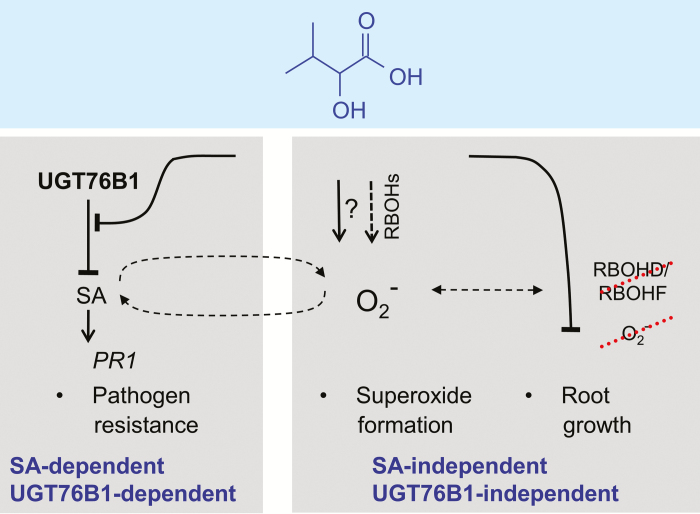
Three separable effects of ILA on *Arabidopsis* plants. ILA activates plant defense and *PR1* marker gene expression in an SA-dependent and UGT76B1-dependent manner. This is attributed to the inhibitory effect of ILA on the UGT76B1-catalysed SA glucosylation. In contrast to the effects of ILA on plant defense, SA and UGT76B1 are not required for the ILA-induced inhibition of root growth and formation of superoxide, since both effects are also found in NahG *sid2* and *ugt76b1* loss-of-function mutants. NADPH oxidases, specifically RBOHD and RBOHF, contribute to O_2_^−^ production; however, this still occurs independently of RBOHs. The ILA-induced inhibition of root growth is neither linked to RBOHD/RBOHF nor blocked by pharmacologically suppressing superoxide formation.

SA-dependent defense responses are triggered by the recognition of pathogens. This leads to enhanced superoxide and hydrogen peroxide production as well as to SID2-dependent SA biosynthesis. Formation of ROS and SA biosynthesis are further activated by a positive feedback mechanism. On the other hand, the level of SA has to be controlled to prevent sustainable damage to the plant (Vlot *et al.*, 2009). A metabolic pathway to control and attenuate the SA response involves the conjugation of the signaling molecule to sugars, catalysed by UDP-carbohydrate-dependent SA glucosyltransferases. SA glucosylation and SA conjugation with amino acids may also suppress the response by initiating SA catabolism (Dempsey *et al.*, 2011). Three enzymes, UGT76B1, UGT74F1, and UGT74F2, glucosylate SA *in vitro* and *in vivo* (Dean and Delaney, 2008; Song *et al.*, 2008; von Saint Paul *et al.*, 2011; Noutoshi *et al.*, 2012; George Thompson *et al.*, 2017). UGT76B1, however, also catalyses the glucosylation of ILA, which, in turn, competitively inhibits SA glucosylation of UGT76B1. Thus, the ILA-dependent enhanced defense response and pathogen resistance can be attributed to the suppression of the attenuation of SA glucosylation. Consequently, ILA can directly affect defense in a UGT76B1-dependent manner. Indeed, the synergistic effect of exogenously added ILA on defense gene expression is lost concomitantly with the loss of the target enzyme UGT76B1 in the *ugt76b1* knockout. Furthermore, the effect of ILA on defense is also SA-dependent, in agreement with such a model. Thus, ILA constitutes a positive regulatory module to reinforce the SA pathway (Fig. 9). ILA declines after infection by *P. syringae*, whereas *UGT76B1* is transcriptionally induced (von Saint Paul *et al.*, 2011; Maksym *et al.*, 2018). Thus, both measures cooperate to enhance UGT76B1-dependent SA conjugation in order to attenuate and control the defense response. In addition, the ILA-enhanced defense could invoke a UGT76B1-independent component through the ILA-induced formation of superoxide (see below). Regardless of the mechanistic implementation, the positive impact of ILA on SA responses constitutes an additional layer of control on the SA-dependent defense pathway by this BCAA-related small-molecule.

### Root growth inhibition by isoleucic acid

Exogenously applied SA, but also other plant hormones such as ABA (Rodrigues *et al.*, 2009), auxin (Okumura *et al.*, 2013) or methyl-jasmonate (Staswick *et al*., 1992), reduced primary root growth. Furthermore, the amino acids proline and glutamic acid repressed root growth of *Arabidopsis* seedlings dependent on SA signaling and a calcium-mediated oxidative burst (Chen *et al.*, 2011). Similar to the synergistic interaction of ILA and SA in reinforcing plant defense, root growth inhibition was enhanced in response to the combination of both substances (Fig. 5C). However, the effect of ILA on root growth differs from its impact on plant defense. While the ILA-enhanced defense is completely lost by *ugt76b1* and dependent on UGT76B1, ILA inhibits root growth of both the wild type and *ugt76b1* mutant plants (Figs 2, 5C). Thus, the root phenotype does not require UGT76B1 (Fig. 9). However, UGT76B1 had a modulating effect and higher expression levels of UGT76B1 attenuated the effect of ILA (Fig. 5C). This can be attributed to the scavenging activity of UGT76B1 lowering the level of unconjugated ILA by glucosylation, thereby diminishing the suppression of root growth. Consequently, ILA itself or a metabolite derived from it other than an ILA glucoside seems to be active in root growth repression. Since Ile also inhibits root growth, a conversion of ILA to Ile could be involved. ILA application led to the specific accumulation of the endogenous Ile level (Maksym *et al.*, 2018). In another study, the exogenous application of 100 µM isoleucine was sufficient to inhibit the root growth of *Arabidopsis* wild type and more strongly of *hdh1* mutant plants, which are defective in Ile degradation (Schertl *et al.*, 2017). On the other hand, *lib1* mutants containing half the wild-type level of Ile showed a reduced root growth as well, i.e. an opposite Ile-related phenotype, which, in turn, was rescued by the addition of only 5 µM of exogenous Ile (Yu *et al.*, 2013*b*). Thus, a deficiency in Ile may repress root growth due to a limitation of the amino acid, whereas a surplus of Ile may have an inhibitory effect as well. Conversely, the Ile-induced root growth inhibition could be mediated by a transformation of Ile into ILA. Currently, the mechanism of root growth inhibition by ILA remains elusive. However, major hormone pathways are not involved, since the effect of ILA on root growth was independent of SA when endogenous SA was eliminated (Fig. 6A), or independent of JA, ABA, and ethylene when employing mutants affecting their biosynthesis or perception (see Supplementary Fig. S6).

### Superoxide formation as a separate effect of isoleucic acid

Both plant defense and root growth are affected and regulated by ROS (Wrzaczek *et al.*, 2013; Tsukagoshi, 2016; Waszczak *et al.*, 2018), which accordingly could provide a link between these effects of ILA. Indeed, ILA induced the formation of superoxide radicals in both leaves and roots, whereas there was no up-regulation of H_2_O_2_.

In *Arabidopsis*, 10 isoforms of NADPH oxidases are known. Only RBOHD and RBOHF are expressed throughout the plant and are crucial for the initial apoplastic generation of superoxide during defense responses (Orman-Ligeza *et al.*, 2016; Morales *et al.*, 2016). O_2_^−^ disproportionation leads to H_2_O_2_ formation, and eventually, SA and ROS form a self-amplifying feedback loop to induce defense. However, several observations support the conclusion that ILA-induced O_2_^−^ formation was a specific and separate effect rather than a major trigger of defense. Firstly, ILA-induced superoxide formation occurs independently of SA. Consequently, the ILA-induced O_2_^−^ is not part of an SA–ROS amplification loop, although it may interfere with the overall cellular redox status. Secondly, superoxide will not be reinforced when ILA is applied together with SA; thereby, it contrasts with the defense response, which is synergistically enhanced by ILA and SA.

The superoxide level is constitutively elevated in *ugt76b1* mutants in conjunction with the enhanced endogenous ILA in these plants. Thus, there is a sustainable shift in the redox balance obviating an immediate signaling function of the enhanced superoxide. Nevertheless, the constitutively higher superoxide level may still interfere with defense reactions. Similar constitutive changes in redox balance due to enhanced superoxide and/or hydrogen peroxide levels have been observed, e.g. upon ectopic expression of the apoplastic PEROXIDASE 57 in *ohy1* mutants leading to an enhanced permeability of the leaf cuticle or in *cpr5* mutants having lost the regulatory nuclear envelope protein CPR5 (Bowling *et al.*, 1997; Survila *et al.*, 2016; Wang *et al.*, 2017). In both scenarios, pathogen defense is affected, yet in different ways. While *ohy1* is more susceptible to virulent *P. syringae*, *cpr5* is more resistant. These divergent outcomes indicate that the actual localization and nature of ROS species will be important. In the case of ILA, the source of superoxide radicals and a potential link of ILA-generated ROS and defense have yet to be clarified. NADPH oxidases, in particular the major isoforms RBOHD and RBOHF, participate in O_2_^−^ formation, but they are not required for its induction by ILA, since (i) *rbohd rbohf* double mutants still show a strong relative induction of superoxide after ILA application and (ii) NBT staining was not abolished by co-incubation of ILA and the RBOH inhibitor DPI (Figs 4, 9). Further elucidation of these processes in plants may be achieved by mechanistic research into the pathology of human maple syrup urine disease or other BCAA-catabolic genetic disorders and *vice versa*, since there are parallels in cellular responses. Importantly, the administration of 2-keto-3-methyl pentanoic acid, the precursor of LA, to the lateral ventricle of rat brains induced several oxidative stress parameters, yet the mechanistic details are not known (Taschetto *et al.*, 2017). In another pathological context, mitochondrial superoxide formation induced by branched-chain keto acids was correlated with cardiac dysfunction (Sun and Wang, 2016).

ROS formation affects root elongation by controlling both the root meristem size and the transition from cell division to elongation (Tsukagoshi, 2016). Opposing gradients of O_2_^−^ and H_2_O_2_ occur in the root tips with a higher O_2_^−^ level in the root meristem of wild type plants, whereas H_2_O_2_ is enhanced in the differentiation zone. The balance of superoxide and hydrogen peroxide controls the transition zone and thereby determines the size of the meristem versus the positioning of the elongation zone (Dunand *et al.*, 2007; Tsukagoshi *et al.*, 2010). Mutants, transgenic lines, or pharmacological treatments altering this shift by affecting peroxidases or NADPH oxidases lead to altered root growth; suppression of H_2_O_2_ formation and enhanced O_2_^−^ levels lead to longer roots and vice versa (Foreman *et al.*, 2003; Tsukagoshi *et al.*, 2010; Tsukagoshi, 2016). ILA enhances superoxide in the meristem and differentiation/elongation zone and even in mature root tissues. Thereby, it will interfere with the wild type redox balance by expanding the O_2_^−^-dominated region in conjunction with the unchanged H_2_O_2_ level. This change in superoxide content and distribution could be the reason why ILA represses root growth instead of promoting it. On the other hand, several findings argue against a causal link of the ILA-induced O_2_^−^ in inhibiting root growth. Firstly, the *rbohd rbohf* double mutant lacking two important oxidases still shows root growth inhibition like the wild type. Kwak *et al.* (2003) had shown that RBOHD and RBOHF were involved in ABA-induced root growth inhibition. Thus, the independence of ILA-induced growth suppression of ABA further supports the notion that RBOHD and RBOHF are not involved. Secondly, the inhibition is still observed in the presence of an O_2_^−^ scavenger. Furthermore, Ile and LA show similar root growth inhibition like ILA, but they are not able to induce superoxide radicals indicating a different and possibly even common mechanism of ILA, LA, and Ile in suppressing root growth independent of superoxide as a primary cause. Taken together, the inhibition of root growth by ILA could be separated as an SA- and UGT76B1-independent response as well as a primarily O_2_^−^-independent effect.

### Specificity of isoleucic acid perception and action in comparison with leucic acid and isoleucine

The 2-hydroxycarboxylic acid ILA affects distinct processes in plants, which can be separated into SA-dependent and SA-independent as well as UGT76B1-dependent and UGT76B1-independent responses of *Arabidopsis*. Due to its chemical structure and evidence provided by Maksym *et al*. (2018), ILA is likely linked to the metabolism of the BCAA isoleucine, although this has not yet been unequivocally demonstrated. Nevertheless, ILA obviously joins a group of amino acid-related small molecules and signaling compounds in plants such as γ-aminobutyric acid or pipecolic acid (Mekonnen *et al.*, 2016; Hartmann and Zeier, 2018). ILA is differentially accumulating among BCAA-related 2-hydroxycarboxylic acids (Maksym *et al.*, 2018). In addition to the differential accumulation of LA and ILA, the effects of ILA are specific and distinct from those of LA and the related amino acid Ile in *Arabidopsis*. Therefore, the perception of ILA seems to occur at multiple levels: while UGT76B1 could be the target in relation to SA-dependent defense, the perception mechanisms leading to the induction of superoxide formation and to the inhibition of root growth are elusive. However, such unknown mechanisms differentiate ILA from the closely related isomeric LA and from the amino acid analog Ile.

## Supplementary data

Supplementary data are available at JXB online.

Fig. S1. ILA competitively inhibits SA glucosylation by UGT76B1.

Fig. S2. Effect of ILA on the expression of the SA marker gene *PR1* in leaves of 14-day-old wild type and NahG *sid2* plants grown in liquid culture.

Fig. S3. Interaction of SA and ILA in wild type at higher SA concentrations.

Fig. S4. SA-dependent induction of *PR1* in *ugt76b1*.

Fig. S5. Induction of ROS in ILA-treated plants.

Fig. S6. Root growth inhibition by ILA of different hormone-related mutants.

Fig. S7. Root growth inhibition by Ile, LA, and ILA.

Table S1. List of primers used for quantitative real-time PCR.

eraa160_suppl_Supplementary_Figures_S1_S7_Table_S1

## Abbreviations

DAB: 3,3′-diaminobenzidine;
ILA: isoleucic acid
LA: leucic acid
NBT: nitroblue tetrazolium
SA: salicylic acid
UGT: UDP carbohydrate-dependent glycosyltransferase
WT: wild type
