# Supplementary material for: The isoleucic acid triad: distinct impacts on plant defense, root growth, and formation of reactive oxygen species
Source: J Exp Bot. 2020 Mar 28;71(14):4258–70. doi: 10.1093/jxb/eraa160 (PMC7448199; doi:10.1093/jxb/eraa160)
Supplement: eraa160_suppl_Supplementary_Figures_S1_S7_Table_S1 [file eraa160_suppl_supplementary_figures_s1_s7_table_s1.pdf]

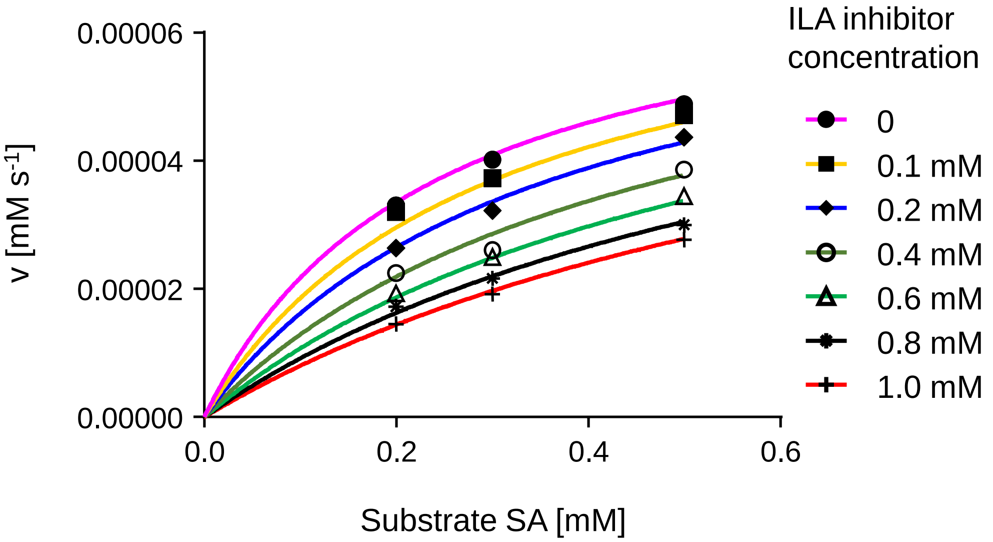

Fig. S1. ILA competitively inhibits SA glucosylation by UGT76B1. The qualitative inhibition of UGT76B1-catalyzed SA glucosylation by ILA had been shown by Noutoshi et al. (2012). Here, recombinant UGT76B1 was used to assess the type of inhibition. SA glucoside formation was determined using 0.5 µg GST-UGT76B1 fusion protein (von Saint Paul et al., 2016) with 0.2 mM, 0.3 mM or 0.5 mM SA and 5 mM UDP glucose in the presence of increasing ILA concentrations (30 °C, 30 min). SAG formation was quantified by HPLC separation and UV absorbance at 302 nm. Data were plotted and analyzed by GraphPad Prism 6 (GraphPad Software, San Diego, CA, USA) indicating a competitive inhibition with a K_i = 410 µM ± 33 µM (SE).

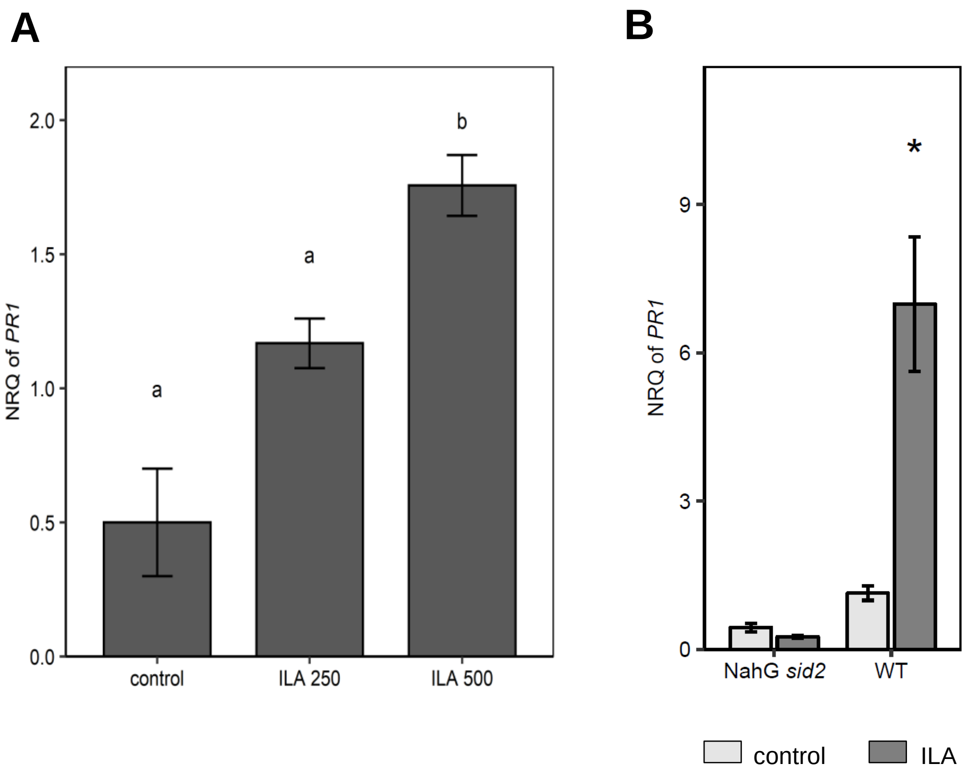

Fig. S2. Effect of ILA on the expression of the SA marker gene *PR1* in leaves of 14-day-old plantlets grown in liquid culture. (A) Transcript abundance of *PR1* was measured by RT-qPCR 48 h after application of 250 or 500 µM ILA to wild-type plants. Gene expression was normalized to *S16* and *UBQ5*; means ± SE; n = 3 – 4. Significant differences (p.adj. values) are indicated by letters according to one-way ANOVA. (B) Transcript abundance of *PR1* assessed by RT-qPCR analysis 48 h after application of 500 µM ILA to wild-type (WT) or SA-depleted NahG *sid2* plants. Gene expression was normalized to *S16* and *UBQ5*; means ± SE; n = 4. Differences between treated or untreated plants were analyzed by Welch two sample t-test; * = $p < 0.05$.

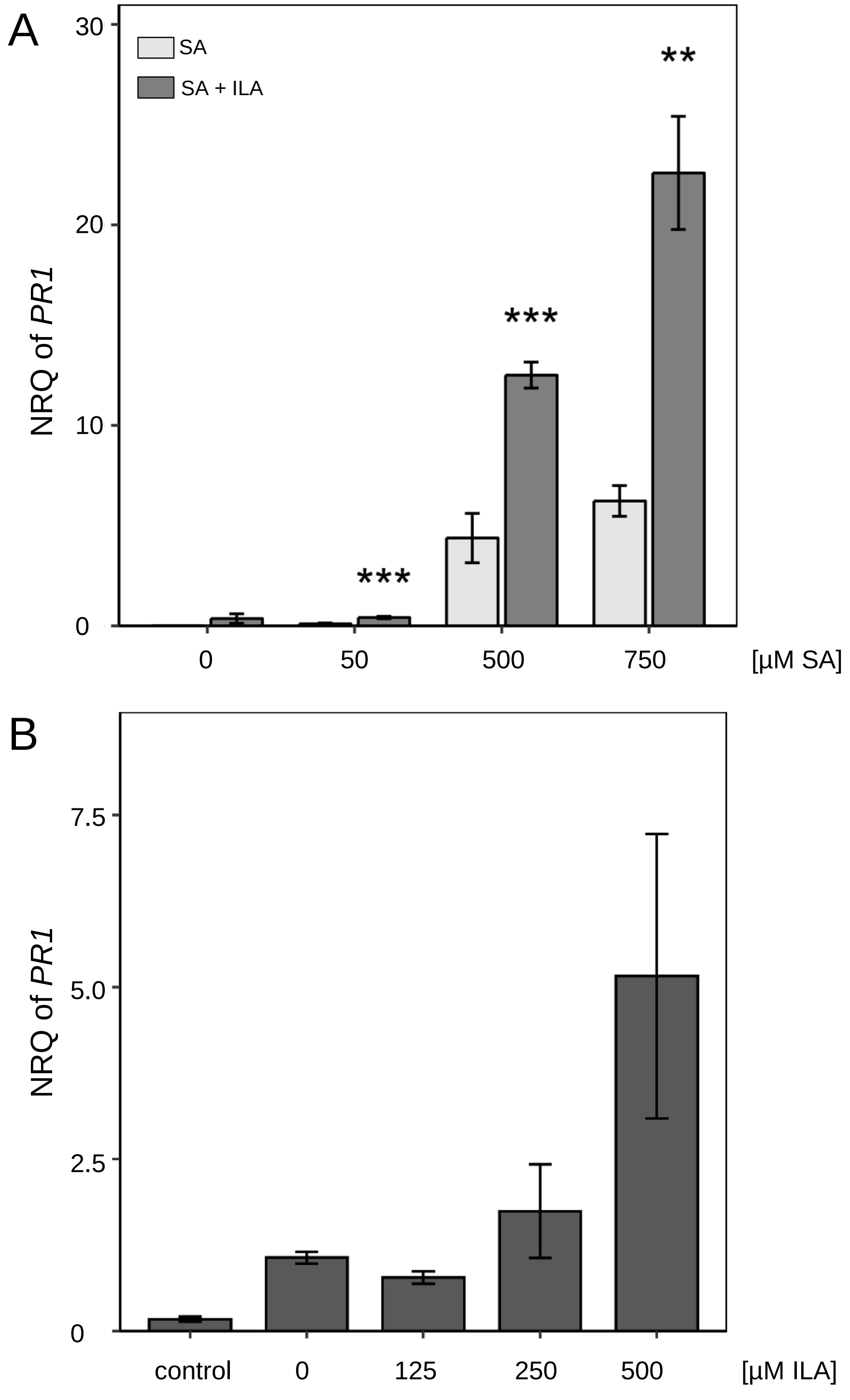

Fig. S3. Interaction of SA and ILA in wild type. (A) The interaction was assessed at higher, increasing SA concentrations. *PR1* expression in leaves of 14-day-old wild-type seedlings 48 h after addition of media with increasing concentrations of SA without and with ILA (250 µM). Seedlings were either treated with SA medium (light grey bars) or medium containing 250 µM ILA in addition to SA (dark grey bars). (B) The interaction of ILA and SA at fixed SA and variable ILA concentrations. Increasing levels of ILA were combined with 100 µM SA. The control experiment lacked both ILA and SA. In both cases, *PR1* expression was normalized to *S16* and *UBQ5*. Means ± SE.; n = 3. The relative expression values may vary in different experiments (compare Fig. 3) due to biological variability of the basal *PR1* expression level. Differences between ILA treated or untreated plants were analyzed by Welch two sample t-test; ** = $p < 0.01$; * = $p < 0.05$.

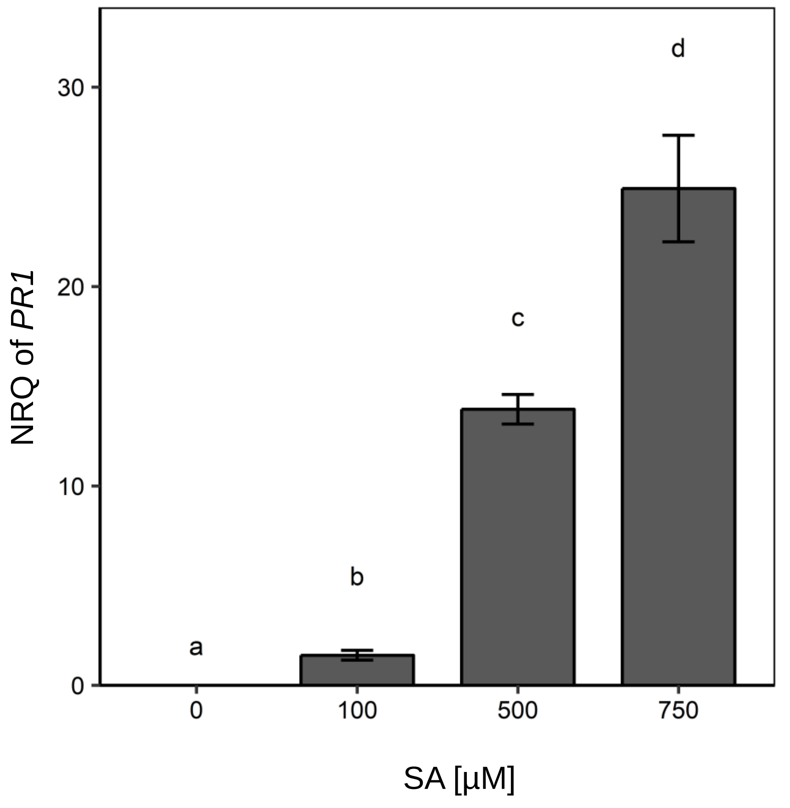

Fig. S4. SA-dependent induction of *PR1* in *ugt76b1* knockout plants. *PR1* expression in leaves of 14-day-old *ugt76b1* seedlings 48 h after addition of media with increasing concentrations of SA as indicated. *PR1* is increasingly induced with higher concentrations of SA. *PR1* expression was normalized to *S16* and *UBQ5*. Means ± SE; n = 4. Significant differences (adjusted p-value < 0.05) are indicated by letters according to one-way ANOVA.

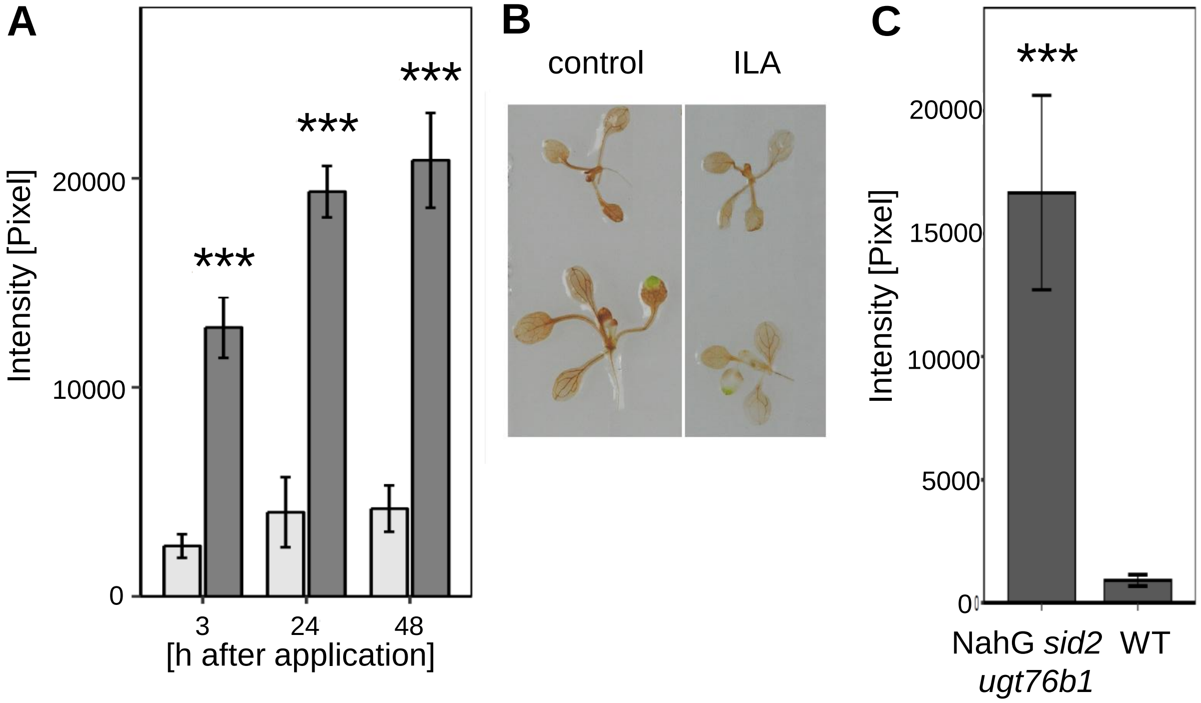

Fig. S5. Induction of ROS in ILA-treated plants. (A) Twelve-day-old plants were treated with 500 µM ILA (dark grey vs. light-grey controls without ILA) and harvested at different time points after addition of ILA. O_2^- radicals were detected using NBT and staining of leaves was quantified. Means ± SE; n = 12. Differences between treated or untreated plants were analyzed by Welch two sample t-test; *** = p < 0.001. (B) Hydrogen peroxide detection in 10-day-old plants grown on control or 500 µM ILA plates. Plants were stained for 8 h with DAB. (C) O_2^- radicals detected in leaves from 14-day-old plants of NahG *sid2 ugt76b1* and wild type (WT) using NBT staining. Means ± SE; n = 18. Differences between genotypes were analyzed by Welch two sample t-test. *** = p < 0.001.

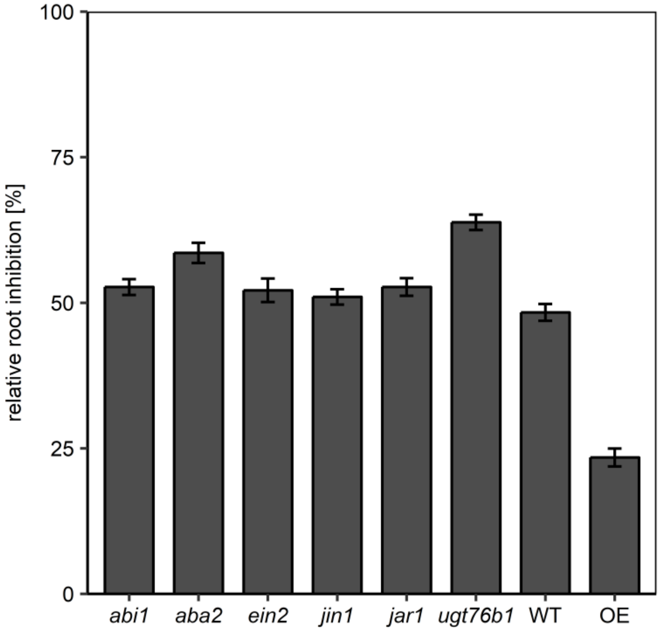

Fig. S6. Root growth inhibition by ILA of different hormone-related mutants. Root growth inhibition after growth for ten days on 500 µM ILA-containing medium is shown relative to root growth on control medium of the respective line. Plant lines related to abscisic acid biosynthesis and perception: *aba2*, *abi1*; to ethylene signaling: *ein2*; and to jasmonic acid signaling and perception: *jin1, jar1* were used. Control and reference lines: *ugt76b1*, wild type (WT) and UGT76B1 overexpressor (OE). n = 11-24.

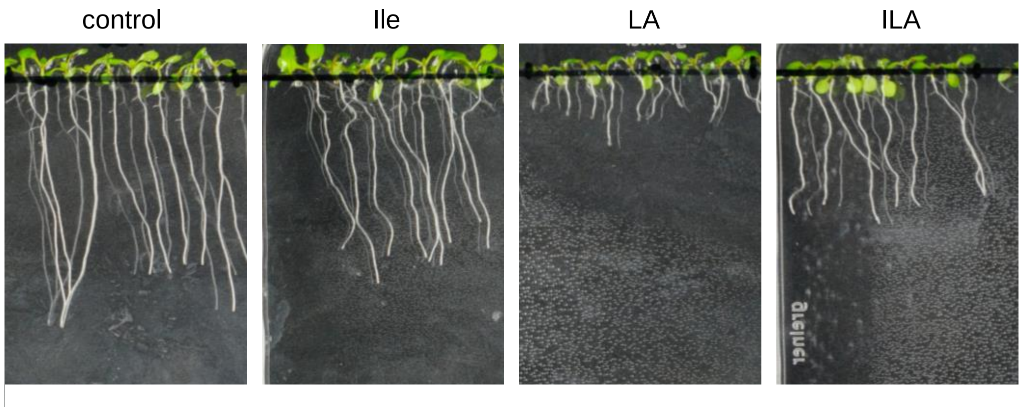

Fig. S7. Root growth inhibition of wild-type plants by Ile, LA and ILA. Root growth inhibition on Ile-, LA- and ILA-containing media (250 µM each). Root lengths were recorded after nine days and are analyzed in Fig. 8C. Since LA already induced a strong root growth inhibition at the concentration of 250 µM, while 500 µM almost inhibited any growth (not shown), the lower concentration was chosen for this experimental series with single compounds applied in contrast to 500 µM as in other experiments.

Table S1. List of primers used for quantitative real-time PCR.

Arabidopsis thaliana genes				
**Gene**	**AGI code**	**Oligonucleotide (forward)**	**Oligonucleotide (reverse)**	**Reference**
UBQ5	At3g62250	5'-GGTGCTAAGAAGAGGAAGAAT	5'-CTCCTTCTTCTGGTAAACGT	von Saint Paul *et al*., 2011
S16	At5g18380, At2g09990	5'-TTTACGCCATCCGTCAGAGTAT	5'-TCTGGTAACGAGAACGAGCAC	von Saint Paul *et al*., 2011
PR1	At2g14610	5'-GTGCCAAAGTGAGGTGTAACAA	5'-CGTGTGTATGCATGATCACATC	von Saint Paul et al., 2011
SAG13	At2g29350	5'-TTGCCCACCCATTGTTAAA	5'-GATTCATGGCTCCTTTGGTT	von Saint Paul *et al*., 2011
CRK7	At4g23150	5'-ATGTCTTCTCTCTTCCCTTTCATATTCC	5'-ACGAGGATCTAAATCAGACATTG	Yeh *et al*., 2015
RBOHF	At1g64060	5'-CTGCGGTTTCGCCATTC	5'-TGTTTCGTCGGCTCTG	Ding *et al*., 2015
RBOHD	At5g47910	5'-ATGATCAAGGTGGCTGTTTACCC	5'-ATCCTTGTGGCTTCGTCATGTG	Mehterov *et al*., 2012
Brassica napus genes				
**Gene**	***Arabidopsis* ortholog (annotation)**	**Oligonucleotide (forward)**	**Oligonucleotide (reverse)**	**Reference**
UP1	At4g33380 (Unknown protein)	5'-AGCCTGAGGAGATATTAGCAGGAA	5'-ATCTCACTGCAGCTCCACCAT	Chen et al., 2010
UBC9	At4g27960 (Ubiquitin con-jugating enzyme 9)	5'-GCATCTGCCTCGACATCTTGA	5'-GACAGCAGCACCTTGGAAATG	Chen et al., 2010
PR1	At2g14610 (Pathogenesis-related protein 1)	5'-AAAGCTACGCCGACCGACTACGAG	5'-CCAGAAAAGTCGGCGCTACTCCA	Alkooranee et al., 2015

References

Alkooranee J., Yin Y, Aledan TR, Jiang Y, Lu G, Wu J, Li M. (2015). Systemic resistance to powdery mildew in *Brassica napus* (AACC) and *Raphanus alboglabra* (RRCC) by *Trichoderma harzianum* TH12. PloS One, **10**, e0142177.

Chen X, Truksa M, Shah S, Weselake RJ. (2010). A survey of quantitative real-time polymerase chain reaction internal reference genes for expression studies in *Brassica napus*. Analytical Biochemistry **405**, 138-140.

Ding S, Zhang B and Qin F. (2015). Arabidopsis RZFP34/CHYR1, a ubiquitin E3 ligase, regulates stomatal movement and drought tolerance via SnRK2. 6-mediated phosphorylation. The Plant Cell, **27**, 3228-3244.

Mehterov N, Balazadeh S, Hille J, Toneva V, Mueller-Roeber B and Gechev T. (2012). Oxidative stress provokes distinct transcriptional responses in the stress-tolerant atr7 and stress-sensitive l*oh2 Arabidopsis thaliana* mutants as revealed by multi-parallel quantitative real-time PCR analysis of ROS marker and antioxidant genes. Plant Physiology and Biochemistry, **59**, 20-29.

von Saint Paul V, Zhang W, Kanawati B, Geist B, Faus-Kessler T, Schmitt-Kopplin P, Schäffner AR. 2011. The Arabidopsis glucosyltransferase UGT76B1 conjugates isoleucic acid and modulates plant defense and senescence. The Plant Cell **23**, 4124-4145.

Yeh YH, Chang YH, Huang PY, Huang JB and Zimmerli L. (2015). Enhanced *Arabidopsis* pattern-triggered immunity by overexpression of cysteine-rich receptor-like kinases. Frontiers in Plant Science, **6**, 322.
